# Supplementary material for: Non-communicable diseases (NCDs) and vulnerability to COVID-19: The case of adult patients with hypertension or diabetes mellitus in Gamo, Gofa, and South Omo zones in Southern Ethiopia
Source: PLoS One. 2022 Jan 25;17(1):e0262642. doi: 10.1371/journal.pone.0262642 (PMC8789109; doi:10.1371/journal.pone.0262642)
Supplement: S2 File — (ZIP) [file pone.0262642.s002.zip › Amharic Version questionnaire.docx]

**ክፍል 1. ማሀበራዊና ነባራዊ ሁኔታ በተመለከተ**

| ተቁ | ዝርዝር | አይነት | ማሳለፍ |
| --- | --- | --- | --- |
| 001 | ጾታ | ወንድ …………….1  ሴት………………....2 |  |
| 002 | ዕድሜ | ________________ |  |
| 003 | ሃይማኖት | ኦርቶዶክስ ……..… 1  ፕሮቴስታንት…………2  ሙስሊም ……………3  ሌላ ካለ ……………....4 |  |
| 004 | የትዳር ሁኔታ | ያላገባ/ች……………... 1  ያገባ/ች ………………. 2  የፈታ/ች ……………… 3  የትዳር አጋር የሞተበት/ባት...4 |  |
| 005 | የሥራ ዓይነት | አርሶአደር…………………1  ተማሪ ……….…………..2  ነጋዴ…………………… .3  የመንግስት ሰራተኛ……….4  የግል ሰራተኛ …………….5  ሌላ ………………………..6 |  |
| 006 | የትምህርት ደረጃ | መጻፍ እና ማንበብ የማይችል………... 1  መጻፍ እና ማንበብ የሚችል ………… 2  የመጀመሪያ ደረጃ ያጠናቀቀ (1-8) ……..3  ሁለተኛ ደረጃ ያጠናቀቀ (9-12)…...….. 4  ሴርተፊከት እና በላይ …………….…….5 |  |
| 007 | መኖሪያ ከተማ | አርባምንጭ … 1 ሳውላ………2  ጅንካ ከተማ ....……………….…3 |  |
| 008 | ወራዊ ገቢ |  |  |

| **ክፍል 2 : ስለ COVID-19 እዉቀት በተመለከተ** | | | |
| --- | --- | --- | --- |
| CN | ስለ ኮሮና ምልክቶች በተመለከተ | **ነዉ** | **አይደለም** |
| 2.1 | የኮሮና በሽታ ዋና ዋና ምልክቶች ትኩሳት፤ድካም ፤ደረቅ ሳልና የጡንቻ ህመም ናቸዉ |  |  |
| 2.2 | የአፍንጫ መታፈን፤ንፍጥ እና ማስነጠስን የመሳሰሉ የጉንፋን ምልክቶች እምብዛም የኮሮና በሽታ ምልክቶች አይደሉም |  |  |
|  | **ስለኮሮና ተጋላጭነት እዉቀት በተመለከተ** |  |  |
| 2.3 | በኮሮና ቫይረስ የተያዘ ሰዉ ሁሉ በከፍተኛ ደረጃ ይታመማል ማለት አይደለም፡ ነገር ግን እድሜያቸዉ የገፋ፡የቆየ በሽታ ያለባቸዉ ፤እና ከመጠን በላይ ወፍራም ሰዎች በሽታዉ ይጠናባቸዋል። |  |  |
| 2.4 | የኮሮና ቫይረስ ፍቱን መድሃኒት የለዉም።ነገር ግን ምልክቶችን በማከም ብዙዎች ከበሽታዉ ይፈወሳሉ። |  |  |
|  | **ስለበሽታዉ መተላለፊያ መንገድ እዉቀት በተመለከተ** |  |  |
| 2.5 | የኮሮና ቫይረስ በመተንፈሻ አካል አማካኝነት በትንፋሽ ይተላለፋል። |  |  |
| 2.6 | ከዱር እንሰሳትጋር መነካካት ወይም ስጋቸዉን መመገብ ለኮሮና ቫይረስ ያጋልጣል። |  |  |
| 2.7 | ትኩሳት እስከሌለዉ ድረስ በኮሮና ቫይረስ የተያዘ ሰዉ ለሌላዉ ሰዉ ማስተላለፍ አይችልም። |  |  |
|  | **ስለመከላከያ መንገዶች** |  |  |
| 2.8 | እጅን በዉሃና ሳሙና በደንብ መታጠብ አንዱ የኮረና ቫይረስ መከላከያ መንገድ ነዉ። |  |  |
| 2.9 | ባልታጠበ እጅ አይንንና አፍንጫን አለመነካካት አንዱ የኮሮና ቫይረስ መከላከያ መንገድ ነዉ። |  |  |
| 2.10 | የኮሮና ቫይርስን ስርጭት ለመከላከል፤ሰዎች በተሰበሰበበት ማለትም በባቡር ጣቢያዎች፤የህዝብ ትራንስፖርት ምጠቀም የለባቸዉም። |  |  |
| 2.11 | የኮሮና ቫይረስ ስርጭትን ለመከላከል ማንኛዉም ሰዉ የህክምና ማስኮችን መጠቀም አለበት። |  |  |
| 2.12 | በኮሮና ቫይረስ ከተያዘ ሰዉ ጋር ንኪኪ ያለዉ ሰዉ ወድያዉኑ ተለይቶ መቀመጥ አለበጥ። |  |  |
| 2.13 | በኮሮና ቫይረስ የተጠቁ ሰዎችን ለይቶ ማቆያና ህክምና ክፍል ዉስጥ ማቆየት የቫይረሱን ስርጭት ለመቀነስል ይረዳል። |  |  |
| 2.14 | ልጆችና ወጣቶችን ከኮሮና ቫይረስመከላከልእምብዛም አስፈላጊ አይደለም። |  |  |

ክፍል:3፡ የኮቭድ-19 መከላከያ መንገዶችን በሚመለከት

| ተቁ | ዝርዝር | ዓይነት | ማሳለፍ |
| --- | --- | --- | --- |
| 3.1 | እጅዎን በሳሙናና በውሃ በተከታታይ ይታጠባሉ | አዎን ----------------------1  አይደለም ------------------2 |  |
| 3.2 | በሰላምታ አሰጣጥቶ ላይ እጅ መጨባበጥዎን አስዎገዱ? | ኦዎን ----------------------1  አይደለም ---------------- 2 |  |
| 3.3 | በሰላምታ አሰጣጥቶ ርቀትዎን ጠብቀዋል | ኦዎን ----------------------1  አይደለም ------------------2 |  |
| 3.4 | ሰው ወደበዛበት ቦታ ሄደው ያውቃሉ? | ኦዎን ----------------------1  አይደለም ---------------- 2 |  |
| 3.5 | ከቤትዎ ሲወጡ የአፍንጫ ና አፍ መሸፈኛ ተጠቅመዋል? | ኦዎን ----------------------1  አይደለም ------------------2 |  |
| 3.6 | ሲያስልዎትና ስያስነጥስዎት አፍንጫ እና አፍዎን ይሸፍናሉ? | ኦዎን ----------------------1  አይደለም ------------------2 |  |
| 3.7 | በቤትዎ ይቆዩን ተግብረዋል? | ኦዎን ----------------------1  አይደለም ------------------2 |  |
| 3.8 | ሳኒታይዘር ይጠቀማሉ? | ኦዎን ----------------------1  አይደለም ------------------2 |  |
| 3.9 | የስኳር/ደም ግፊት ታማሚዎች ከሌሎች በተለየ ለኮቭድ19 ተጋላጭ መሆናቸውን ያውቁ ኖሯል? | ኦዎን ----------------------1  አይደለም ------------------2 |  |
| 3.10 | ለጥያቄ ተራ ቁ 17 መልስዎ አዎ ከሆነ ለተጋላጭነታቸው መረጃ ከየት ነው ያገኙት? | ከጤና ባለሙያ -----------1  ከቤተሰብ -----------------.2  ከጓደኛ --------------------3  ከማህበራዊ ሚዲያ ------4  ሌላ -----------------------5 |  |

| **ክፍል 4 : የስኳር/የደም ግፊት ህክምና ክትትልን በተመለከቴ** | | | |
| --- | --- | --- | --- |
| 4.1 | የበሽታ አይነት ይጥቀሱ | 1. የስኳር በሽታ 2. የደም ግፊት |  |
| 4.2 | የስኳር/የደም ግፍት እንዳለቦዎት መቼ ነዉ ያወቁት | ----------------------- |  |
| 4.3 | የስኳር/የደም ግፊት ህክምና መቼ ጀመሩ | _________ |  |
| 4.4 | ለስኳር /ለደም ግፊት ህክምና ክትትል ያደርጋሉ | 1. አዎ 2. አይደለም |  |
| 4.5 | ኮሮና በሽታ ከመከሰቱ በፊት/ የህክምና ክትትል/check-up/ መቼ መቼ ያደርጋሉ | 1. በየወር 2. በየሁለት ወር 3. በየሶስት ወር 4. በየሰድስት ወ ር 5. የህመም ስሜት ስሰማኝ ብቻ 6. ክትትል/ check-up/ አድርጌ አላዉቅም 7. ሌላ ካለ ይጥቀሱ______ |  |
| **4.6** | ለጥያቄ ተ.ቁ 23መልስ ክትትል/ check-up/ አድርጌ አላዉቅም ከሆነ ለምን | - - - 1. ጠናዬ ላይ መሻሻል ስለታዬ       2. የህክምና ገንዘብ ስሌለኝ       3. ህክምና ቦታ ስለምርቅ       4. ህክምና ቦታ ብዙ ሠዓት መጠበቅ ስለምጠላ       5. ሌላ ካለ ይጥቀሱ______ |  |
| **4.7** | ኮሮና በሽታ ከተከሰተ በኃላ / የህክምና ክትትል/check-up/ አድርገዉ ያዉቃሉ | 1.አዎ  2.አላዉቅም |  |
| **4.8** | ለጥያቄ ተ.ቁ 25 መልስዎ ክትትል/ check-up/ አድርጌ አላዉቅም ከሆነ ለምን | - - - 1. ጠናዬ ላይ መሻሻል ስለታየ       2. የህክምና ገንዘብ ስለለኝ       3. ህክምና ቦታ ስለምርቅ       4. ህክምና ቦታ ብዙ ሠዓት መጠበቅ ስለምጠላ       5. በኮሮና በሽታ እነዳልያዝ ፈርቼ       6. ለላ ካለ ይጥቀሱ______ |  |
| **4.9** | የመጨረሻ ህክምና ክትትል መቼ ነዉ ያደረጉት/ይጥቀሱ/ | ______________ |  |
| **4.10** | መዲኃኒት በአግባቡ ይወስዳሉ | 1. አዎ 2. አልወስድም 3. አቋርጫለሁ 4. ሌላ ከለ ይጥቀሱ________ |  |
| **4.11** | ለ ጥያቄ ተ.ቁ.28 መልስ አቋርጫለሁ ከሆነ መቼ/ይጥቀሱ/ | ______________________ |  |
| **4.12** | ለ ጥያቄ ተ.ቁ.28 መልስዎ አቋርጫለሁ ከሆነ ያቋረጡበት ምክንያት /ይጥቀሱ/ | 1. ህክምና ቦታ ስለምርቅ 2. በኮሮና በሽታ እንዳልያዝ ፈርቼ 3. ስለተሻለኝ 4. ህክምና ገንዘብ ስለለኝ 5. ሌላ ካለ ይጥቀሱ___________ |  |
| **4.13** | ለ ጥያቄ ተ.ቁ.28 መልስ አቋርጫለሁ ከሆነ ጤናዎትን ለመጠበቅ ምንድነዉ የምያደርጉት | 1. ስፖርት እሠራለሁ 2. ባህላዊ ህክምና እከታተላለሁ 3. ምንም አላደርግም 4. ሌላ ካለ ይጥቀሱ________ |  |
| **ክፍል : አምስት የስኳር/የደም ግፊት በሽታ መቆጣጠርን በተመለከቴ** | | | |
| **5.1** | የስኳር/የደም ግፊት በሽታን እንደት መቆጣጠር ይቻላል | 1. በአግባቡ መድኃንት በመዉሰድ 2. አመጋገባችን በማስተካከል 3. የኑሮ ዜይብያችን በማስተካከል 4. ሌላ ካለ ይጥቀሱ--------------- |  |
| **5.2** | የስኳር /የደም ግፊት በሽታን ለመቆጣጠር የእርሶን የአመጋገብ ሁኔታ ምን ይመስላል | 1. ፍራፍረና አትክልት    1. በየቀኑ እመገባለሁ    2. አልፎ አልፎ እመገባለሁ    3. በፍጹም አልወስድም 2. እንስሳትና የእንስሳት ዉጤቶች    1. በየቀኑ እመገባለሁ    2. አልፎ አልፎ እመገባለሁ    3. በፍጹም አልወስድም |  |
| **5.3** | የስኳር /የደም ግፊት በሽታ እንዳለቦዎት ካወቁበት ጊዜ ጀምሮ በሽታዉን ለመቆጣጠር ምን አይነት የኑሮ ዜይበ ይከተላሉ | 1. ስጋራ ማጨስ አቁሚያለሁ 2. አልኮል መጠጣት አቁሚያለሁ 3. ከጨዉ ነጻ የሆነ ምግብ መጠቀም 4. ስፖርት መስራት 5. ሌላ ካለ ይጥቀሱ__________ |  |
