# Supplementary material for: Non-communicable diseases (NCDs) and vulnerability to COVID-19: The case of adult patients with hypertension or diabetes mellitus in Gamo, Gofa, and South Omo zones in Southern Ethiopia
Source: PLoS One. 2022 Jan 25;17(1):e0262642. doi: 10.1371/journal.pone.0262642 (PMC8789109; doi:10.1371/journal.pone.0262642)
Supplement: S2 File — (ZIP) [file pone.0262642.s002.zip › English version questionnaire.docx]

| **Code** | Variables | Category | Skip |
| --- | --- | --- | --- |
| **001** | Sex | Male …………………….1  Female ………………….2 |  |
| **002** | Age | …………………… |  |
| **003** | Religion | Orthodox …………….…1  Protestant ………………2  Muslim …………………3  Others ………………….4 |  |
| **004** | What is your Marital Status | Single ……………….….1  Married ……………....... .2  Divorce ………………… 3  Widowed ……………..…4 |  |
| **005** | Occupation | Farmer……………………1  Student ………………...…2  Currently unemployed…....3  Government employee …...4  Private/business employee...5 |  |
| **006** | What is the highest level of education you have attained | Cannot read and write …….1  Read and write ……………2  Primary (1–8 )……………..3  Secondary (9–12).………….4  College and above………… 5 |  |
| **007** | Town | Arba Minch………………1  Sawla ………………….…2  Jinika …………………….3 |  |
| **008** | Monthly income (ETB) | ____________ |  |

**Part I: Socio-demographic and economic characteristics.**

| **Part II: COVID-19 knowledge related** | | | |
| --- | --- | --- | --- |
| CN | Knowledge of symptoms | **cor** | **NC** |
| 2.1 | The main clinical symptoms of COVID-19 are fever, fatigue, dry cough, and myalgia. |  |  |
| 2.2 | Unlike the common cold, stuffy nose, runny nose, and sneezing are less common in persons infected with the COVID-19 virus. |  |  |
|  | **Knowledge of high risk** |  |  |
| 2.3 | Not all persons with COVID-19 will develop severe cases. But only those who are elderly, have chronic illnesses, and are obese are more likely to develop severe cases. |  |  |
| 2.4 | There is no effective cure for COVID-19, but early symptomatic and supportive treatment can help most patients recover from the infection. |  |  |
|  | **Knowledge about Mode of transmissions and infectiousness** |  |  |
| 2.5 | The COVID-19 virus spreads via the respiratory droplets of an infected individual. |  |  |
| 2.6 | Eating or contacting wild animals would result in infection by the COVID-19 virus. |  |  |
| 2.7 | Persons with COVID-19 cannot infect the virus to others when a fever is not present. |  |  |
|  | **Knowledge about ways of prevention** |  |  |
| 2.8 | Proper washing hands with soap and water is one method of preventing COVID-19. |  |  |
| 2.9 | One way of prevention of COVID 19 is not touching the eye and nose with unwashed hands. |  |  |
| 2.10 | To prevent the infection by COVID-19, individuals should avoid going to crowded places such as train stations and taking public transportations. |  |  |
| 2.11 | Ordinary residents can wear general medical masks to prevent the infection by the COVID- 19 |  |  |
| 2.12 | People who had contact with someone infected with the COVID-19 should immediately be isolated. |  |  |
| 2.13 | Isolations and treatment of people infected with the COVID-19 virus are effective ways to reduce the spread of the virus. |  |  |
| 2.14 | Children and young adults don’t need to take measures to prevent the infection by the COVID-19. |  |  |
|  | **Quartile s of correctly answered knowledge (of 14 items)** |  |  |
|  | Quartile 1 (0–25%) |  |  |
|  | Quartile 2 [25–50%) |  |  |
|  | Quartile 3 [50–75%) |  |  |
|  | Quartile 4 [75–100%) |  |  |

| **COVID 19 Preventive measures** | | Frequency | |
| --- | --- | --- | --- |
|  |  | Yes | No |
| **3.1** | Do you frequently wash your hands with water and soap? |  |  |
| **3.2** | Do you stopped shaking hands while giving a greeting |  |  |
| **3.3** | Did you avoid proximity, including while greetings (within1m)? |  |  |
| **3.4** | have not been going to crowded places |  |  |
| **3.5** | Do you wear a face mask when leaving home |  |  |
| **3.6** | Do you avoid touching the eye, nose, mouth before washing hands? |  |  |
| **3.7** | Do you use cover /elbow for coughing/sneezing |  |  |
| **3.8** | have you started to stay home |  |  |
| **3.9** | Do you use (alcohol-rubbing, no contact with surfaces) |  |  |

**Part III COV ID-19 Preventive measures**

| **Part IV: Knowledge of Vulnerability to COVID -19** | | | |
| --- | --- | --- | --- |
| **Code** | **Category** | **Variable** | **Remark** |
| **4.1** | People with DM/HTN are more vulnerable to develop severe forms of COVID- 19 than other relatively healthy people. | Yes…………1  No………….2 |  |
| **4.2** | Source of information for knowledge vulnerability | Health professionals……….1  Family……………………...2  Friends……………………...3  Mass-media……………… ...4  Other, specify------------------.5 |  |

| **Part V: status of adherence to DM/HTN** | | | |
| --- | --- | --- | --- |
| **Code** | **Category** | **Variable** | **Skip** |
| 5.1 | When have you been diagnosed | ________ |  |
| 5.2 | When did you start treatment? | _________ |  |
| 5.3 | Do you have a healthcare follow-up? | Yes…….1 No……2 |  |
| 5.4 | How often do you visit a health facility for a checkup? | Every moth…………………….1  Every two months………….….2  Every three months….................3  Every six months……………....4  When I feel symptoms worsen…5  Other (Specify,)_____………….6 |  |
| **5.5** | When was your last recent follow-up? | ______________ |  |
| **5.6** | Do you take your prescribed medication regularly | Yes..............................................1  No………………………….…...2  Discontinued…………………...3  Other (Specify)_______……….4 |  |
| **5.7** | **If no/discontinued**, when did you stop your DM/HTN medications? | ______________________ |  |
| **5.8** | I**f no/discontinued** reason for not taking regularly | Distance………………………...1  Fear of acquiring COVID-19…...2  Because I feel better…………….3  Lack of money………………….4  Other (Specify)__________…….5 |  |
| **5.9** | **If no/discontinued**, what alternative measures you are practicing | Exercise………………………. .1  Traditional medicine…………...2  I do nothing…………………….3  Other (Specify)_________....….4 |  |
