# Supplementary material for: Non-communicable diseases (NCDs) and vulnerability to COVID-19: The case of adult patients with hypertension or diabetes mellitus in Gamo, Gofa, and South Omo zones in Southern Ethiopia
Source: PLoS One. 2022 Jan 25;17(1):e0262642. doi: 10.1371/journal.pone.0262642 (PMC8789109; doi:10.1371/journal.pone.0262642)
Supplement: Attachment — Submitted filename: Response to Reviewers(Round 2).docx [file pone.0262642.s004.docx]

**Response to the editor’s and reviewer’s comments**

Dear Editor/Reviewers, we are very glad to have your valuable comments. We have now revised the manuscript based on your comments.

Kind regards,

Aleme Mekuria

Reviewer #1:

Thank you for the important point you raised.

We used data from other countries to verify the finding and discuss the potential biological mechanisms. Therefore,

1. We deeply discussed the biological and pathophysiological mechanisms of both diabetes and hypertension
2. The relationship of these diseases with COVID-19, and
3. How these diseases worsen the clinical outcomes of COVID-19
4. We used nine additional references from other countries to discuss the above points

Please kindly refer to the discussion section of the revised manuscript on page 20, line number 372-388 (the first two paragraphs) and the reference section (ref. no 22-30).

Reviewer # 2

1. We have corrected/edited grammatical and sentence structure errors across the document using online grammar editing software to edit the spelling, grammar, and language usage. Additionally, we used a professional English language editor to check for grammar and sentence structure errors. Therefore, we provided a much-improved manuscript.
2. We have included the reason for setting the cut-off point, 2500 ETB for the monthly income variable in the document. Please kindly refer to page 8, line 220-228.
3. We have corrected each table for the format, alignments, and typographical errors.

Thank you all for your commitment to evaluate our manuscript!
