# Supplementary material for: Non-communicable diseases (NCDs) and vulnerability to COVID-19: The case of adult patients with hypertension or diabetes mellitus in Gamo, Gofa, and South Omo zones in Southern Ethiopia
Source: PLoS One. 2022 Jan 25;17(1):e0262642. doi: 10.1371/journal.pone.0262642 (PMC8789109; doi:10.1371/journal.pone.0262642)
Supplement: Attachment — Submitted filename: response to reviewers.docx [file pone.0262642.s005.docx]

Dear reviewer, thank you for providing the references. We have read them and found they are important. However, most of them are experimental study designs to identify the genetic causal link between an outcome variable and factors. However, our study is not about the causal link between diabetes or hypertension and COVID-19. The overall objective of this study was to assess the knowledge and preventive practices of the COVID-9 among patients with hypertension or diabetes. We used a community-based cross-sectional study design with a multi-stage random sampling technique and selected sufficient and representative sample from the three large zones of the Southern region of Ethiopia. Using logistic regression analysis, we have identified several independent predictors of knowledge and preventive practices of COVID-19. The magnitude of the association between the outcome and each independent variable was identified. Based on the study findings, we have forwarded recommendations to adopt major preventive practices to prevent the contraction of the virus. Furthermore, we suggested the Ministry of Health of Ethiopia to give priority to COVID-19 vaccination to these patients with underlying disease conditions.

Thank you so much.

Aleme Mekuria (MPH/RH, PhD. Fellow)
